# Coverage of Second Dose of Measles-Containing Vaccine (MCV-2) and Japanese Encephalitis Vaccine (JE-2) and Its Predictors Among Children 2–5 Years Old in the Ormanjhi Block of Ranchi, Jharkhand, India: A Mixed Method Study

**DOI:** 10.7759/cureus.73004

**Published:** 2024-11-04

**Authors:** Ankita Mukul, Shashi B Singh, Vidya Sagar, Dilip K Paswan, Smiti Narain, Dewesh Kumar

**Affiliations:** 1 Department of Community Medicine, Rajendra Institute of Medical Sciences, Ranchi, IND; 2 Department of Community Medicine/Preventive and Social Medicine, Rajendra Institute of Medical Sciences, Ranchi, IND

**Keywords:** japanese encephalitis, je vaccine, mcv, measles, predictors, vaccination coverage

## Abstract

Introduction

Administering the measles vaccine at an appropriate age and dosage in children is important for India to eliminate measles, a potentially deadly vaccine-preventable disease. Similarly, the Japanese encephalitis (JE) vaccine, particularly in endemic regions is important to prevent morbidity and high-case fatality from the disease. The study attempts to evaluate the coverage of measles and JE vaccines and their predictors.

Materials and methods

A mixed method study design, incorporating a three-stage cluster random sampling process was used in the cross-sectional survey conducted among 604 children aged 2-5 years in the Ormanjhi block of Ranchi, Jharkhand, from April 2023 to June 2024. The parents/caregivers of the eligible children were interviewed using a predesigned, pretested semi-structured questionnaire, and the immunization status of children was taken from vaccination cards. A focused group discussion (FGD) and an in-depth interview (IDI) with healthcare providers formed the qualitative component. We conducted descriptive, and logistic regression analysis using the IBM SPSS Statistics for Windows, Version 26 (Released 2019; IBM Corp., Armonk, New York, United States). The association between the coverage of the vaccine and sociodemographic variables was done using the Chi-square test. Logistic regression was used to study the predictors, and a p-value of <0.05 was considered statistically significant.

Results

In the household survey, the coverage of the measles-containing vaccine first dose (MCV-1) was 594 (98.3%), while the second dose (MCV-2) was 536 (88.7%) out of 604 participants. For the JE vaccine, the coverage of the first dose (JE-1) was 588 (97.4%), while the second dose (JE-2) coverage was 492 (81.5%). In the multivariate logistic regression, religion and the lack of parental knowledge about measles were significant predictors for lower MCV-2 uptake, while the for JE vaccine, religion, father's education, and household head's occupation remained statistically significant factors (p ≤ 0.05). Vaccine hesitancy had mixed perceptions with the child’s unavailability being the most significant reason for hesitancy in both MCV and JE vaccinations. In the FGD, child’s unavailability at times of vaccination and parent’s fear of side effects post vaccination were major factors for missing doses of the vaccine.

Conclusion

While the initial coverage of MCV and JE vaccines is commendable, the substantial drop in the second dose coverage and the delays in administration present significant challenges.

## Introduction

Immunization forms a critical component of primary health care and ensures the nation's health security by protecting children from serious preventable diseases. In India with a population of more than one billion and more than 25 million new births every year, immunizing each birth cohort for all the vaccine antigens is a mammoth task where vaccine-preventable diseases are still prevalent [1,2,3]. A strong and well-planned immunization program that lowers the number of vaccine-preventable diseases is key to meeting Sustainable Development Goal (SDG)-3, to reduce the infant mortality rate (IMR) to 25 per 1000 live births by 2030. The current IMR of India is 28, while that of Jharkhand is 25 per 1000 live births as per SRS 2020 [4].

Measles is one of the leading vaccine-preventable causes of child mortality and morbidity worldwide as unimmunized young children face the highest risk of severe complications. Herd immunity is crucial, as unvaccinated individuals remain vulnerable and can spread the disease further in the community [5]. In India, measles accounts for 2% of all under-five mortalities [5,6]. While measles vaccination has reduced global deaths by 73% between 2000 and 2018, the disease remains common in many developing countries, especially in Africa and Asia [7]. Coverage at or above 95% with two doses of measles-containing vaccine (MCV) is recommended to ensure long-term immunity and address primary vaccine failure, as about 15% of vaccinated children fail to develop immunity following the first dose of MCV (MCV-1) [7,8]. By 2018, 86% of the world's children received their first measles vaccine by age one, up from 72% in 2000, but only 69% received the second dose [7]. The National Family Health Survey (NFHS-5) (2019-2021) data shows that 87.9% of Indian children aged 12-23 months received the MCV-1 dose, but only 31.9% of those aged 24-35 months received the second dose of MCV (MCV-2). In Jharkhand, these figures are 86.7% for MCV-1 and 32.3% for MCV-2 [9]. In 2018, of the 19.2 million infants unvaccinated with at least one dose of MCV, approximately 6.1 million were from India, Nigeria, and Pakistan, while the highest measles cases in 2019 were reported in Madagascar, Ukraine, India, Nigeria, Kazakhstan, Chad, Myanmar, Thailand, and the Philippines [10]. Ongoing vigilance is crucial to maintain progress against measles, as outbreaks can be lethal particularly in the face of natural disasters like the recent COVID-19 [11] pandemic which disrupted healthcare and delayed the introduction of the second dose in many places.

Japanese encephalitis (JE) is another mosquito-borne flavivirus disease affecting young children (<15 years age group), causing acute encephalitis syndrome (AES) with long-term neuropsychiatric sequelae. It has been reported in India as an epidemic and sporadic encephalitis from around 171 districts of 19 states. While the overall incidence of JE is 1.8 per 100,000 per year in endemic countries, it is 5.5 among 1-15-year-old children [12]. Vaccination against JE virus is a cornerstone for JE control and prevention. Vaccine efficacy is reported to be between 80% and 99% following single-dose vaccination and ≥98% with two doses [12,13]. Over the past few decades, JE incidence has decreased considerably in some countries (Japan, South Korea, and Taiwan) but has increased in others including India [14]. The first dose of JE vaccine (JE-1) during 9-12 months and the second dose of JE vaccine (JE-2) during 16-24 months of age are administered under the National Immunization Schedule (NIS) in endemic districts along with MCV [14]. In addition to adequate knowledge of JE and a positive perception of JE vaccine for adoption of preventive measures, high coverage of the JE vaccine in populations at risk of disease is required to reduce the JE burden.

Despite the proven efficacy of these vaccines, achieving and maintaining high coverage rates for the MCV-2 and JE-2 vaccines remains a significant public health challenge [14]. The Ormanjhi block of the Ranchi district in India presents a unique context for studying vaccination coverage as it comprises of rural and semi-urban populations. This area, with its socioeconomic and geographic characteristics, faces distinct challenges in healthcare delivery, including immunization services. Frequent outbreaks of measles have been reported from few places in the Ormanjhi block of Ranchi. There is ample data describing coverage with the MCV-1 and JE-1 vaccine. However, little information is available about the coverage of MCV-2 and JE-2 vaccine which are key to measles elimination and JE control efforts. Local predictors and barriers must be understood to improve vaccination rates in specific blocks like Ormanjhi, where detailed studies are lacking despite substantial research on national and state levels. So, a study was conducted with the aim of learning the coverage of MCV-2 and JE-2 and its predictors in the Ormanjhi block of Ranchi, Jharkhand. The primary objective of the study was to determine the coverage of MCV-2 and JE -2 vaccination in the study area, and secondary objectives were to find the corelates of measles and JE vaccination coverage, to assess vaccine hesitancy amongst the parents/caregivers of the beneficiaries of these vaccines, and to plan and develop an intervention to improve the coverage of MCV-2 and JE-2 vaccines based on the study findings.

## Materials and methods

Study setting and duration

The research study was carried out in the Ormanjhi block in the Ranchi district of Jharkhand, from July 1, 2022, to June 30, 2024 (24 months), which is also the catering area of the Rural Health Training Center of Rajendra Institute of Medical Sciences (RIMS), Ranchi. The total population of the Ormanjhi block is more than one lakh.

Study design

The study employed a mixed method study design that combined community-based cross-sectional survey and qualitative studies in the form of focused group discussions (FGDs) with auxiliary nurse midwives (ANM) and accredited social health activists (ASHA; also known as Sahiyas in Jharkhand) along with in-depth interviews (IDIs) with medical officers of health centers in Ormanjhi.

For cross-sectional household survey

Study Population

Parents/caregivers of children aged 2-5 years (henceforth called eligible children) were the population selected to participate in the cross-sectional household survey.

Eligibility Criteria

Children aged 2-5 years belonging to the area and parents/guardians giving their consent to participate in the study were included.

Sample Size and Sampling Technique

Based on the NFHS-5 data conducted in 2019-2021 [9], the percentage of children aged 24-35 months who received the MCV-2 was 31.9%; the sample size was calculated using the formula N = z2 pq/d2, taking the standard normal deviation (Z) of 1.96 for a 95% confidence interval and absolute precision (d) of 5%, proportion (p) of children vaccinated with MCV-2 as 32% and proportion (q) of children unvaccinated with MCV-2 as 68%. To increase the validity of the study, a design effect of 1.5 and 10% nonresponse rate were applied. Therefore, the final sample size required for the study was calculated as 600. In our study, we covered 604 children to meet our objectives. The data was collected using the three-stage cluster random sampling method to sample 604 children (2-5 years). The Ormanjhi block comprises of 26 subcenters and two primary health centers (PHCs) making a total of 28 centers. Out of this, 1/3rd of centers, i.e., around 10 health centers (PHCs and subcenters) were selected by simple random sampling method. From each health center, a list of Anganwadi centers (AWCs) was obtained, and the ASHAs catering to the villages under the AWC was approached to make a line list of all eligible children’s households. From each health center, 4-5 villages were selected, with each village representing one cluster, so there were total 40 clusters. From each village/cluster, 15 eligible children were recruited consecutively. If there were not enough eligible children from one village, the next adjacent village was chosen to complete the number. So, in this way, the requisite sample size for the study was achieved. The parent or guardian of the child available at the time of the survey was interviewed, preferably mother of the child. If in any household there were more than one eligible child, then the parents were interviewed about all the children as individual child.

Data Collection Tool and Techniques

Data collection was carried out for one year at the household level by a house-to-house survey where children in the age group of 2-5 years were present. The parents/guardians of eligible children, available at the time of the survey were interviewed face to face using a predesigned, semi-structured, pretested study proforma, after obtaining written informed consent in the Hindi language from the parents. The study proforma included three sections, namely, sociodemographic section, immunization section, and vaccine hesitancy section. The questions were administered verbally to the participants, and the response was entered in the proforma by the interviewer. There were no nonresponders. The information about vaccines was gathered from the mother and child protection (MCP) card primarily and, where card was not available, from registers in the AWC or subcenter. 

Data Management and Statistical Analysis

Data analysis was done using IBM SPSS Statistics for Windows, Version 26 (Released 2019; IBM Corp., Armonk, New York, United States). Descriptive statistics were conducted to summarize the distribution of study variables using frequency, percentages, means, and standard deviation where ever appropriate. Association between vaccination coverage and various sociodemographic variables was done using the Chi-square test. The independent variables (sociodemographic) associated with children’s vaccination status at p < 0.05 level was analyzed using the logistic regression model, and a value of p < 0.05 was considered statistically significant. Content analysis was used to interpret the findings of qualitative data.

FGDs

Three FGD sessions were carried out with the healthcare providers, namely, the Sahiyas and ANMs catering to the villages and subcenters randomly selected for data collection for the house-to-house survey. One FGD was done with the ANMs of the subcenters, second FGD with the Sahiyas working at the grass-root level, and one FGD was done with Sahiyas working under a particular subcenter where majority of the population belonged to the minority community. The community-specific FGD was planned because majority of the defaulters (dropouts and refusals) for vaccination were found in the particular subcenter only and measles outbreak have been reported from the same lately.

Study Participants and Sample Size

The two FGDs included 10 ANMs and eight Sahiyas from subcenters randomly selected for our study. Third FGD had seven Sahiyas from a particular subcenter (having minority population) with verbal consent obtained beforehand. The FGDs, held sequentially in May 2024, utilized an open-ended FGD guide with some prompts to explore parents’ and caregivers' perceptions regarding childhood immunization, interactions with healthcare workers, challenges in service delivery, and suggestions for improving MCV-2 and JE-2 vaccine coverage. The FGD interview guide was developed in English, translated into Hindi with backtranslation, then validated by experts, and the FGDs were also audio-recorded. The principal researcher facilitated the discussions, while colleagues took notes, capturing key points for qualitative analysis and preparing sociograms for all three sessions. The anonymized audio-recording, originally in Hindi, was transcribed by listening to the audio. The data was condensed into themes and subthemes, followed by triangulating the findings from the FGDs with the results of the household survey. IDIs were conducted with medical officers of the PHC and community health center (CHC) using an open-ended interview schedule with the aim to understand the entire process of vaccine arrangement and delivery to beneficiaries at AWCs, monitoring and supervision of vaccination sessions, challenges faced, gaps in community knowledge, and perceptions about childhood vaccination and gathered suggestions on how to improve vaccine coverage.

## Results

Sociodemographic characteristics of child and caregiver/parents

A total of 604 eligible children were included in the study. The mean age of the child was 38.71 months (±11.85 standard deviation (SD)). The average birth order of children in the study was 1.78 (±0.907 SD) suggesting most children were the first child in their family. The average birth weight of children was 2.7673 kg (±0.45 SD). The average birth interval was 2.43 years (±1.407 SD). Majority of the respondents were the mother of the child, whose average age was 27.34 years (±5.8 SD) (Table 1).

**Table 1:** Characteristic of the study participants in the household survey of children aged 2-5 years in the Ormanjhi block of Ranchi, Jharkhand (N = 604) N: Number/frequency * Modified Brahm Govind (BG) Prasad classification (2023) for socioeconomic status of family

Characteristic	Categories	N	Percentage
Sociodemographic determinants			
Age of child (completed months)	24-35 months	274	45.4
	36-47 months	180	29.8
	>47 months	150	24.8
Gender of child	Male	314	52.0
	female	290	48.0
Birth weight of child	<2.5 Kg	230	38.1
	≥2.5 Kg	374	61.9
Religion	Hindu	137	22.7
	Muslim	387	64.1
	Sarna	80	13.2
Caste	General	103	17.1
	Schedule caste	16	2.6
	Schedule tribe	93	15.4
	Other backward class	392	64.9
Ethnicity	Tribal	93	15.4
	Nontribal	511	84.6
Birth order	First born	285	47.2
	Second child	200	33.1
	Third child	89	14.7
	Fourth child	24	4.0
	≥fifth child	6	1.0
Birth interval	First child	283	46.9
	1 year	18	3.0
	2 years	63	10.4
	≥3 years	240	39.7
Place of delivery	Institutional	600	99.3
	Home	4	0.7
Socioeconomic determinants			
*Socioeconomic status of family	Class I	23	3.8
	Class II	112	18.5
	Class III	153	25.3
	Class IV	218	36.1
	Class V	98	16.2
Type of family	Joint	340	56.3
	Nuclear	264	43.7
Mother’s education	Illiterate	29	4.8
	Primary	21	3.5
	Secondary	257	42.5
	Higher secondary/inter	148	24.5
	Graduation	140	23.2
	Post-graduation	6	1.0
	Mother not alive	3	0.5
Father’s education	Illiterate	31	5.1
	Primary	20	3.3
	Secondary	266	44.0
	Higher secondary/ inter	160	26.5
	Graduation	114	18.9
	Post-graduation	4	0.7
	Father not alive	9	1.5
Mother’s occupation	House wife	553	91.6
	Daily wage worker	18	3.0
	Part time farming in own field	3	0.5
	Government job	4	0.7
	Private job	23	3.8
	Mother not alive	3	0.5
Father’s occupation	Unemployed	12	2.0
	Farming	20	3.3
	Shopkeeper	93	15.4
	Daily wage worker	213	35.3
	Government job	21	3.5
	Private job	236	39.1
	Father not alive	9	1.5
Parent’s marital status	Married	592	98.0
	Only one parent alive	12	2.0
Gender of the head of household	Male	538	89.1
	Female	66	10.9
Occupation of head of the household	Agriculture (works in own field)	31	5.1
	Agricultural laborer/daily wage laborer	112	18.5
	Self-employed/small business/artisan	163	27.0
	Works in shops for salary	15	2.5
	Employed in private companies	86	14.2
	Government employee	17	2.8
	Retired employee	24	4.0
	Home maker	15	2.5
	Unemployed	141	23.3
Caregiver of child	Mother	560	92.7
	Father and others	44	7.3

MCV and JE vaccine coverage from vaccination cards

**Figure 1:**
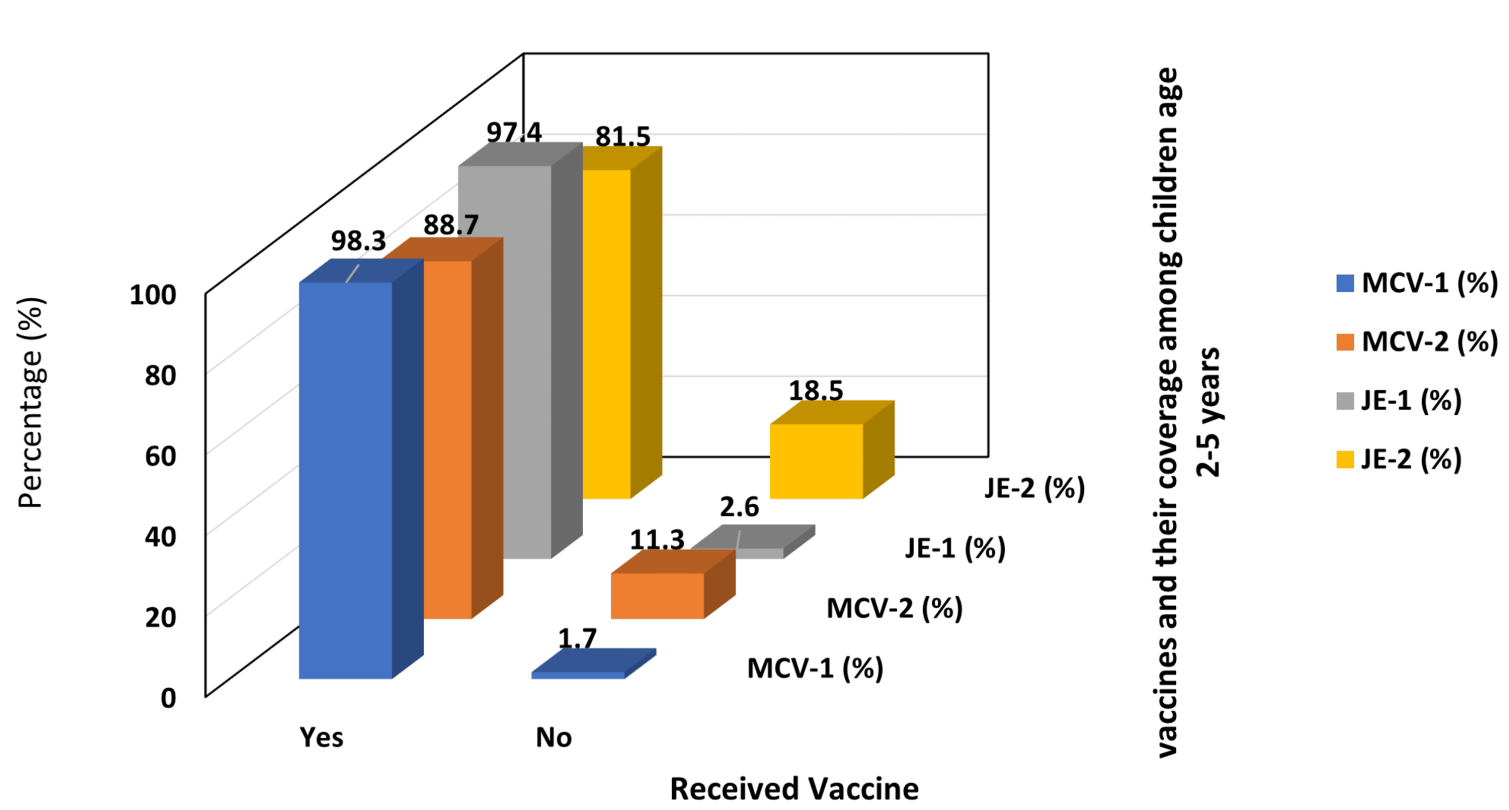
Coverage of the first and second doses of MCV and JE vaccine in children aged 2-5 years in the Ormanjhi block of the Ranchi district (N = 604) MCV-1: Measles-containing vaccine first dose; MCV-2: measles-containing vaccine second dose; JE-1: Japanese encephalitis vaccine first dose; JE-2: Japanese encephalitis vaccine second dose

The overall coverage for MCV-1 was 594 (98.3%), while for MCV-2, it was 536 (88.7%) out of 604. The coverage for JE-1 vaccine was 588 (97.4%), while for the JE-2 vaccine, it was 492 (81.5%) (Figure 1).

Additionally, in our study, out of the total of 604 subjects, 536 (88.7%) of the children were MCV completely vaccinated, while 58 (9.6%) of the children were MCV partially vaccinated, and 10 (1.7%) of the children were MCV not vaccinated. Similarly for JE vaccine, 492 (81.5%) were JE completely vaccinated, a notable 96 (15.9%) were JE partially vaccinated, and 16 (2.6%) of children were JE not vaccinated. For the MCV series, a substantial 369 (61.1%) of the children received MCV-1 late (mean delay of 72.5 days), and an alarming 495 (82%) received MCV-2 late (mean delay of 107.34 days). Similarly, for the JE vaccine series, 371 (61.4%) of the children were delayed in receiving JE-1 (78.09 days), and 454 (75.2%) with JE-2 (99.44 days) (refer to Appendix for definition).

Factors associated with less uptake of MCV-2 and JE-2 vaccine

In the multivariate logistic regression for MCV, Muslim religion, parents’ lack of knowledge about measles, and children belonging to class III socioeconomic status (Modified Brahm Govind (BG) Prasad, 2023 classification) were found to be statistically significant with less uptake of MCV-2 (p ≤ 0.05) (Table 2).

**Table 2:** Results of the multivariate logistic regression for factors associated with less uptake of measles-containing vaccine second dose (MCV-2) (N = 604) S: Statistically significant; *: reference group; N: number/frequency; AOR: adjusted odds ratio; CI: confidence interval A p-value of <0.05 was considered as a statistically significant result

Characteristics	Categories	N	AOR	95% CI for AOR		p-value
				Lower	upper	
Religion	Sarna*	80	1.000	-	-	-
	Hindu	137	1.045	0.293	3.730	0.946
	Muslim	387	3.411	1.180	9.859	0.023(S)
Socioeconomic status (modified BG prasad scale, 2023)	Class I and Class II *	135	1.000	-	-	-
	Class III	153	2.154	1.037	4.476	0.040(S)
	Class IV and Class V	316	1.049	0.515	2.137	0.896
Knowledge about measles	Yes*	238	1.000	-	-	-
	No	366	0.400	0.236	0.677	0.001(S)

Meanwhile, for JE-2 vaccine, Muslim religion, occupation of the head of household, and father’s education had statistically significant association with less uptake of JE-2 vaccine (p ≤ 0.05) (Table 3).

**Table 3:** Results of the multivariate logistic regression for factors associated with less uptake of the Japanese encephalitis vaccine second dose (JE-2) in children aged 2-5 years in the Ormanjhi block of Ranchi, Jharkhand N: Number/frequency; S: statistically significant; *: reference group; AOR: adjusted odds ratio; CI: confidence interval; HOH: head of household A p-value of <0.05 was considered a statistically significant result

Characteristics	Category		N	AOR	95% CI for AOR		p-value
					Lower	upper	
Religion (N = 604)		Sarna*	80	1.000	-	-	-
		Hindu	137	1.656	0.635	4.324	0.303
		Muslim	387	3.819	1.645	8.867	0.002(S)
Occupation of HOH (N = 604)		Daily wage/agriculture*	306	1.000	-	-	-
		Employed jobs	257	0.929	0.539	1.602	0.792
		Unemployed	32	0.409	0.205	0.815	0.011(S)
Father’s education (N = 595)		Illiterate-primary*	51	1.000	-	-	-
		Middle	118	0.344	0.126	0.936	0.037(S)
		Higher	426	0.997	0.460	2.158	0.993

Vaccine hesitancy

**Figure 2:**
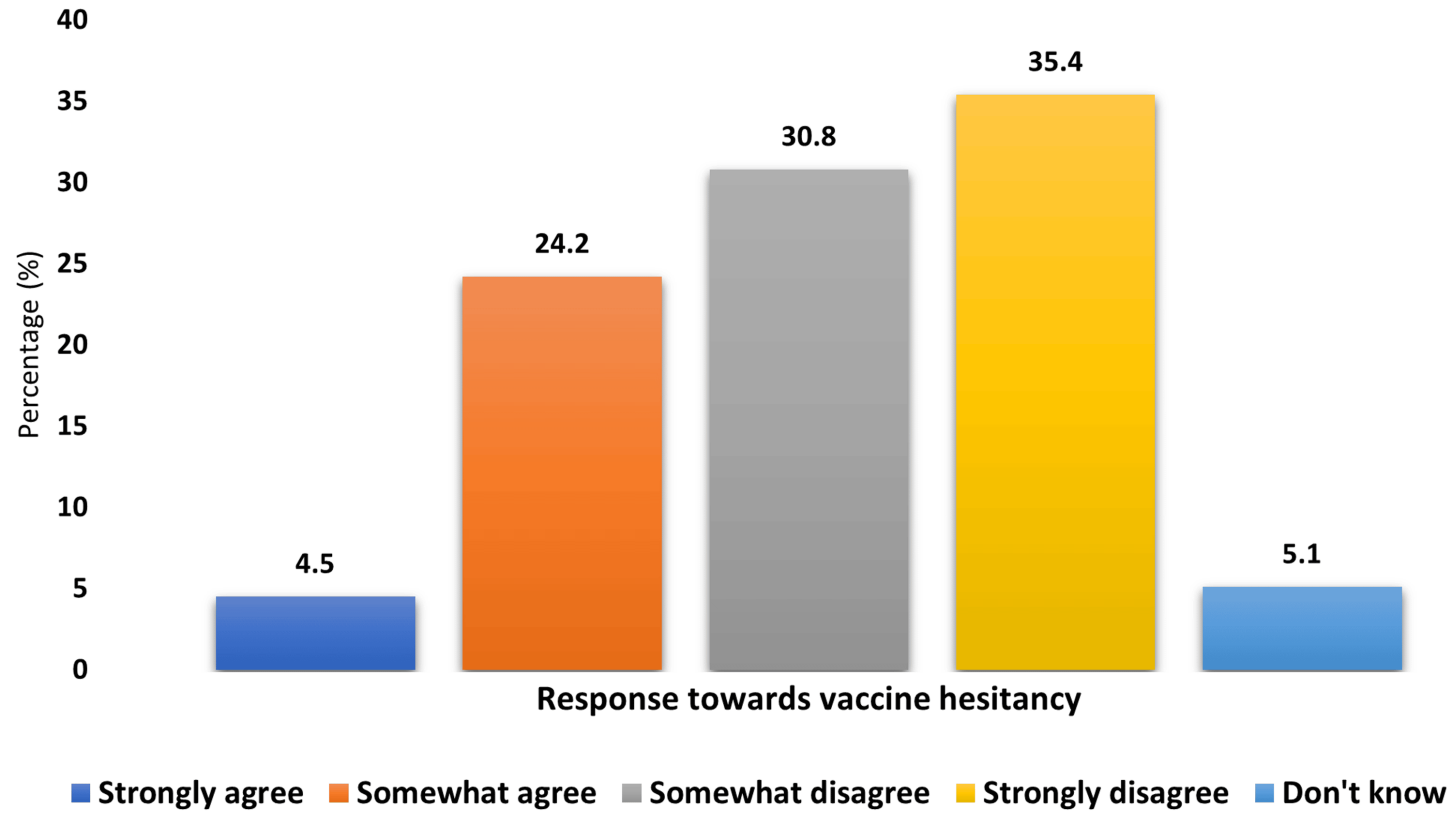
Vaccine hesitancy for routine immunization among parents/caregivers of children aged 2-5 years in the Ormanjhi block (N = 604)

Parents or caregivers showed varied perceptions on vaccine hesitancy, with 400 (66.2%) out of 604 participants disagreeing on vaccine hesitancy statements (Figure 2).

Overall, while the child’s unavailability (either sickness or not being present at place during vaccination time) was the most significant reason for hesitancy or delay in both MCV and JE vaccinations, other notable factors included concerns about side effects and vaccine unavailability for JE and COVID-19-related issues for both MCV and JE vaccines (Table 4).

**Table 4:** Reasons for vaccine refusal/delay for MCV-2 and JE-2 among parents of children aged 2-5 years in the Ormanjhi block of Jharkhand (N = 604) N: Number/frequency; MCV-2: measles-containing vaccine second dose; JE-2: Japanese encephalitis vaccine second dose

Reasons	Categories		Vaccine refusal/delay for MCV-2	
			N	Percentage
Refusal/delay for MCV-2	Child was sick/unavailable (as per mother’s recall)		431	71.4
	Did not know about the second dose of the vaccine		41	6.8
	Due to corona (fear/no vaccination/others)		37	6.1
	Fear of side effects after the first dose		19	3.1
	Schedule is so long that they forgot to get their child's second dose		17	2.8
	Measles vaccine unavailable		4	0.7
	Parent/guardian thinks receiving the first dose is enough		3	0.5
	Child got measles even after giving the first dose		1	0.2
	Total		553	91.6
Vaccinated on time			49	8.1
Vaccinated early			2	0.3
Reasons		Categories	Vaccine refusal / delay for JE-2 vaccine	
			N	Percentage
Refusal/delay for JE-2		Child was unavailable/sick	399	66.1
		Did not know about the second dose of the vaccine	63	10.4
		JE vaccine unavailable	37	6.1
		Due to corona	36	6.0
		Fear of side-effects after the first dose	12	2.0
		Schedule is so long that they forgot to get their child's second dose	10	1.7
		Parent/guardian thinks receiving the first dose is enough	3	0.5
Vaccinated on time			44	7.3

Vaccine acceptance was primarily driven by advice from healthcare workers, particularly Sahiyas and ANMs (Table 5).

**Table 5:** Reasons for vaccine acceptance for MCV-2/JE-2 vaccine (N = 604) N: Number/frequency; MCV: measles-containing vaccine; JE: Japanese encephalitis; JEV: Japanese encephalitis vaccine

Variables	MCV		JE	
	N	Percentage	N	Percentage
I know it is necessary to protect my child from the disease	260	43.0	13	2.2
ANM/Sahiya told me	219	36.3	471	78.0
I believe in govt. policies for vaccination	49	8.1	48	7.9
Everyone was giving their child a vaccine, so I did	35	5.8	33	5.5
It was being given free of cost by the govt.	31	5.1	6	1.0
Don’t know which vaccines are given	-	-	17	2.8
Not vaccinated with MCV/JEV vaccine	10	1.7	16	2.6

Side effects following vaccination with MCV-2/JE-2 vaccine

Among 604 participants, 503 (83.3%) reported side effects after MCV-2 and 459 (76%) reported side effects after JE-2 vaccine. Among those who reported side effects, the most common side effects reported post-MCV-2 administration was fever among 501 (82.9%), and for JE-2 vaccine, fever was reported by 453 (75%) of the participants, followed by pain (Table 6).

**Table 6:** Side effects following MCV/JE administration (N = 604) N: Number; MCV: measles-containing vaccine; JE: Japanese encephalitis vaccine No other side effects other than fever and pain have been reported

Characteristics	MCV		JE vaccine	
	N	Percentage	N	Percentage
Fever	501	82.9	453	75
Pain	2	0.4	6	1
No side effects reported	92	15.2	129	21.4
Not vaccinated with MCV/JE	9	1.5	16	2.6

FGDs and IDIs

A total of 10 ANMs and 15 Sahiyas participated in the three FGDs, each having 7-8 participants and lasted between 30 and 45 minutes. The result was triangulated with the findings from the household survey, after analyzing it thematically. The study highlighted several challenges affecting vaccination coverage at the AWCs. Overcrowding, long wait times, and limited staff lead to missed or delayed vaccinations. One of the ANM said “at my center I am working alone, in one session I have minimum of 40 children, if any child is left in that session that child won't turn up in the next session because they have to wait for so long to get vaccinated.” Migrant families and minor illnesses further contribute to the delays. The ANM from one Health and Wellness Center (HWC) said that “In my area there are drop out children because my area has more children from rented households and those who have migrated from outside." Supply chain issues were minimal, except for a temporary shortage of the JE vaccine as evidenced by one ANM saying, “only for a few months when JE vaccine was not supplied, we don't face any difficulty in indenting vaccines for our sessions." While the government's free vaccine distribution ensures high coverage, awareness issues persist. One of the Sahiyas said that “people here know that if they get their children vaccinated from private facilities, it will be very expensive. The mothers say that they get free vaccines at AWCs so they come here." Acceptance of vaccines is generally high, but concerns about the side effects post-vaccination, particularly within certain minority communities, have led to refusals and delays. One of the ANM said “if the child cries after vaccination, then the parents say that they will not bring their child for next vaccination session." One Sahiya was of the opinion that “most common reasons for dropouts is because of some festivals, or mother went to her paternal home with the child or if the child is sick." Another Sahiya pointed that “during covid pandemic, some parents did not bring their child for vaccination, and those children did not turn up afterwards also." Sahiyas play a vital role in mobilizing parents. One of the participants said that “most of the parents bring their child for vaccination only after calling them, they don't come on their own."

## Discussion

Our study aimed to find the coverage of the second dose of MCV and JE vaccine among children 2-5 years old in the Ormanjhi block of Ranchi, Jharkhand. Our study reported a high coverage of 594 (98.3%) for MCV-1, while the coverage of MCV-2 remained less at 536 (88.7%). The result was significantly higher when compared to the coverage of MCV-1 and MCV-2 reported in the NFHS-5 report for Ranchi, Jharkhand, and India (MCV-1: 82.0% for Ranchi, 87.8% for Jharkhand, and 88.1% for India; MCV-2: 43.9% for Ranchi, dismal 32.8% for Jharkhand, and 32.4% for India) [9]. Our study reported a considerably higher coverage for MCV-2 than those reported in other studies [15-17]. This could be because, in our study, we found that in some places where measles outbreaks have been reported supplementary immunization activities were conducted and many children who had received their second dose in these sessions were counted as second dose of MCV. Similar studies also highlight that the overall coverage for age-appropriate MCV-1 was higher than the MCV-2, just as in our study [18,19].

In our study, for the JE vaccine, 588 (97.4%) received JE-1 and 492 (81.5%) received JE-2 vaccine. Similar findings were reported from a study conducted by Murhekar et al. [13] in Uttar Pradesh, India, and by Tandale et al. [20] in Central India where the JE vaccination coverage reported was higher than the 90% coverage with appropriate timeliness of vaccines in both states. Furthermore, the analogy of low second-dose coverage compared to the first dose is also supported by a similar study by Kshatri et al. [21].

Another significant finding in our study was that the coverage of JE-2 was comparatively less than the coverage of MCV-2 (although both are scheduled at the same time in the NIS). The reason for this disparity in the JE-2 vaccine was the unavailability of the vaccine as evidenced by the triangulation of data in our study, similar to a study in Central India by Tandale et al. [20].

Low nonvaccination rates for MCV and JE vaccine in our study indicate a generally positive trend toward vaccination acceptance among caregivers in the community, facilitated by the Sahiyas at the grass-root level. However, findings from our study also underscore a concerning trend of delayed vaccination among children for MCV and JE vaccine indicating potential gaps in the effectiveness and timeliness of the local immunization program. These findings from our study are consistent with a study conducted by Yang et al. in China as well [22].

Predictors for low vaccination coverage for MCV-2 and JE-2 vaccines

In our study, children belonging to the Muslim religion, parent’s inadequate knowledge about measles, and middle and lower socioeconomic status of the child showed statistical significance with not receiving MCV-2. Similar findings have been observed in different studies conducted in India where community-specific hesitancy or resistance for vaccinations have been observed [5,23-25].

In a study conducted by Tang et al. [26], the children were less likely to get vaccinated on time, when their caregivers had poor knowledge of vaccines, weak confidence in vaccines, or had low satisfaction with vaccination services. In contrast to this study, our study suggests that parents with less knowledge about measles had less odds of not getting their child vaccinated (AOR: 0.400; CI: 0.236-0.677; p = 0.001). These counterintuitive findings in our study suggests that mere knowledge about measles might not be sufficient to drive higher vaccination rates. It is possible that knowledgeable parents might also be more critical or cautious about vaccines, reflecting a subtle understanding that includes concerns or misconceptions about vaccination.

Although the MCV and JE vaccines are given at the same time in the NIS, the findings emphasize the influence of religion, the occupation of the head of household, and the father's education on vaccination coverage of JE-2 vaccine. Muslim children remain significantly more likely to not receive JE-2 (AOR: 3.819; CI: 1.645-8.867; p = 0.002) as was the case with MCV. Religious beliefs and practices can greatly impact health behaviors and attitudes toward vaccination. The significantly higher odds of Muslim children not receiving JE-2 could be due to specific religious or cultural barriers, misconceptions about vaccines (vaccine resistance), or differing levels of trust in healthcare systems. Other associated factors were children from households where the head was unemployed who were significantly more likely to receive JE-2 compared to those where the head of the household were daily wagers or were involved in agriculture (AOR: 0.409; CI: 0.205-0.185; p = 0.011). Households with unemployed heads may face financial constraints, and therefore, they rely on the government for help. Children of fathers with middle education levels were significantly more likely to receive JE-2 compared to those with illiterate or primary education (AOR: 0.344; CI: 0.126-0.939; p = 0.037). In contrast to other studies where mother’s educational status has an impact on the child’s vaccination status [16-18], our study suggests that the educational level of fathers also plays a critical role in health decision-making. Fathers with middle school education likely have balanced health awareness and access to community health initiatives, leading to better vaccination practices than those with higher education who might prioritize private healthcare.

Vaccine hesitancy for MCV and JE vaccines

Our study shows a strong opposition to vaccine hesitancy among the participants with majority, 400 (66.2%) out of 604 subjects disagreeing with vaccine hesitancy statements. In over half of the participants, 431 (71.4%) for MCV-2 and 399 (66.1%) for JE-2 vaccine, the primary reason for delay was the child's unavailability, often due to minor illness or the child not being present at home during vaccination times similar to the study conducted by Rajkumari et al. [5]. On triangulating the data, the child’s unavailability was also the frequently cited reason by Sahiyas and ANMs in the FGDs. Noncompliance of parents toward vaccination schedule was another reason that they have cited in the FGDs. Additionally, vaccine unavailability in 37 (6.1%) was notably significant for the JE vaccine, as supported by information from IDIs and FGDs as well that JE vaccine supply was interrupted for some time in the area. Fear of side effects and concerns related to COVID-19 were common themes across both vaccines.

Reasons for vaccine acceptance for MCV and JE vaccines

In our study, the primary reason for vaccine acceptance for MCV was the parent’s understanding of the necessity of protecting their child from disease (260, 43.0%), followed by health worker’s advice as a significant motivator in 219 (36.3%) participants. However, the predominant reason for accepting the JE vaccine was advice from an ANM or Sahiya, cited by 471 (78.0%) of the respondents, similar to a study by Krishnendhu et al. [24], where the drivers to acceptance of vaccination campaign were the team effort of the healthcare providers.

Side effects post-vaccination with MCV and JE vaccines

Among the 604 participants in our study, 503 (83.3%) reported side effects post-vaccination with MCV, and 459 (76.0%) participants reported side effects post-JE vaccine. The most common side effect was fever which was 501 (82.9%) for MCV and 453 (75.0%) for JE vaccine, with pain being rarely reported, similar to the study done in Nigeria by Gbenewei et al. [27] where out of the total 6,214 suspected cases of AEFI, fever accounted for 38%, while pain at injection site was 30% among the common side effects reported.

Findings from the FGDs and IDIs

Our study identifies key challenges and facilitators for vaccine uptake in the study area. While free vaccines and accessible AWCs enhance coverage, challenges like inadequate staffing, migrant family influx, and space limitations hinder efficiency. Awareness is generally high, but lapses in parental recall and cultural concerns, especially among Muslim families, affect timely vaccination. Sahiyas play a crucial role in activating vaccinations, but there's a need to empower families to take more initiative. In a similar study conducted in India by Francis et al. [28], findings suggests that parents identified difficulties in assessing routine immunization when travelling for work and showed knowledge gaps regarding the benefits and risks of vaccination and fears surrounding certain vaccines due to negative news report and common side effects following childhood vaccinations.

This study is novel in its focus on MCV and JE vaccine coverage in Jharkhand (as limited data is available in this context), using comprehensive data and a mixed method design to enhance reliability and context-specific insights which are its major strength. However, its findings are geographically limited to the Ormanjhi block, may not generalize broadly, and are constrained by a cross-sectional design and limited literature on JE vaccine coverage. Based on the findings, the study recommends targeted interventions to improve vaccination coverage, particularly among low socioeconomic groups and particular communities, and emphasizes making vaccination compulsory before school entry. It suggests enhancing community awareness, increasing resources for health workers, and improving accessibility through more centers and mobile units. Additionally, it advocates for leveraging technology, engaging community leaders, and establishing monitoring mechanisms to address vaccine hesitancy and ensure timely vaccinations. By focusing on these areas, it is possible to improve vaccination rates and move toward the elimination of measles and control of JE in this region.

## Conclusions

Our study provides significant insights into the coverage and predictors of the MCV-2 and JE-2, highlighting several critical aspects of vaccination coverage and its associated sociodemographic factors. While the initial coverage of MCV and JE vaccines is commendable, the substantial drop in the second-dose coverage and the delays in administration remain significant challenges in ensuring complete immunization essential for long-term immunity and herd protection. The delay trend in vaccination indicates systemic issues in vaccine delivery and follow-ups. Thus, the study provides a foundation for further research and interventions aimed at improving public health outcomes in similar settings.

## Appendices

Operational definitions used in the study

For Vaccination Status of Children for MCV/JE Vaccine

Measles/JE completely vaccinated: Those children aged 2-5 years who have received both doses of the MCV/JE vaccine.

Measles/JE partially vaccinated: Those children aged 2-5 years who have been vaccinated with only one dose of the MCV/JE vaccine (i.e., not received the second dose).

Measles/JE not vaccinated: Those children aged 2-5 years who have not received any dose of the MCV/JE vaccine.

Early/timely vaccinated: For measles-containing vaccine (MCV- 1 and MCV-2) and Japanese encephalitis vaccine (JE-1 and JE-2), those children who have received the doses of the vaccine on time (due date of the vaccine + 30 days) or earlier than the due date are called as early/timely vaccination.

Delayed vaccination: For measles-containing vaccine (MCV- 1 and MCV-2) and Japanese encephalitis vaccine (JE-1 and JE-2), those children who have received the doses of the vaccine after the recommended time (for the first dose: nine completed months + 30 days and for the second dose: 15 completed months + 30 days) were chosen as the cutoff day for delayed vaccination.

Vaccine refusal or not vaccinated: Those children who have not received even a single dose of the MCV or JE vaccine are categorized as vaccine refusal.

Vaccine hesitancy: According to the Strategic Advisory Group of Experts (SAGE) and vaccine hesitancy working group of the World Health Organization (WHO), vaccine hesitancy refers to delay in acceptance or refusal of vaccines despite the availability of vaccine services. It is the human behavior that is influenced by 3Cs, i.e., issues of confidence (no trust in vaccine or provider), complacency (does not perceive the need for vaccination, does not value the vaccine), and convenience (ease or difficulty of access).

Vaccine acceptance: Vaccine acceptance is defined as willingness to get vaccinated.

Vaccine resistance: Vaccine resistance is defined as those people who will definitely not be willing to get vaccinated.
